# Lung function and collagen 1a levels are associated with changes in 6 min walk test distance during treatment of TB among HIV-infected adults: a prospective cohort study

**DOI:** 10.1186/s12890-023-02325-7

**Published:** 2023-02-03

**Authors:** Yeonsoo Baik, Pholo Maenetje, Diana Schramm, Caroline Tiemessen, Itai Ncube, Gavin Churchyard, Robert Wallis, Mboyo-di-tamba Vangu, Hardy Kornfeld, Yun Li, Sara C. Auld, Gregory P. Bisson

**Affiliations:** 1 Department of Biostatistics, Epidemiology, and Informatics, Center for Clinical Epidemiology and Biostatistics, Perelman School of Medicine at the University of Pennsylvania, 832 Blockley Hall, 423 Guardian Drive, Philadelphia, PA 19104-6021 USA; 2 The Aurum Institute, Johannesburg, South Africa; 3 Department of Virology, Faculty of Health Sciences, School of Pathology, University of the Witwatersrand, Johannesburg, South Africa; 4 Centre for HIV and STIs, National Institute for Communicable Diseases, Johannesburg, South Africa; 5 School of Public Health, University of Witwatersrand, Johannesburg, South Africa; 6 Department of Nuclear Medicine, CM Johannesburg Academic Hospital, University of the Witwatersrand, Johannesburg, South Africa; 7 Department of Medicine, University of Massachusetts Chan Medical School, Worcester, USA; 8 Department of Medicine, Emory University Rollins School of Public Health and School of Medicine, Atlanta, GA USA; 9 Division of Infectious Diseases, Department of Medicine, Perelman School of Medicine at the University of Pennsylvania, Philadelphia, PA USA

**Keywords:** Tuberculosis, HIV/TB, Six-minute walk test, Inflammatory biomarkers, Post-treatment

## Abstract

**Background:**

Patients with tuberculosis (TB) and HIV often present with impairments in lung function and exercise capacity after treatment. We evaluated clinical and immunologic variables associated with a minimum clinically important difference (MCID) in the change in the 6 min walk test distance during the first 24 weeks of antiretroviral (ART) and anti-tubercular therapy.

**Methods:**

Adults initiating ART and anti-TB treatment in the setting of newly-diagnosed HIV and pulmonary TB were enrolled in a prospective cohort study in South Africa. Patients underwent 6 min walk tests and spirometry at weeks 0, 4, 12, and 24 and biomarker level measurements early during treatment, at weeks 0, 4, and 12, when inflammation levels are typically elevated. Biomarkers included matrix metalloproteinases-1 (MMP-1), tissue inhibitor of MMP (TIMP)-1, collagen 1a, IL-6, IL-8, vascular cell adhesion molecule 1 (VCAM-1), C-X-C motif chemokine 10 (CXCL-10), CXCL-11, macrophage colony-stimulating factor (M-CSF), plasminogen activator, vascular endothelial growth factor, and chemokine (C-C) motif-2 (CCL-2). An MCID was derived statistically, and achievement of an MCID was modeled as the outcome using logistic regression model.

**Results:**

Eighty-nine patients walked an average of 393 (± standard deviation = 69) meters at baseline, which increased by an average of 9% (430 ± 70 m) at week 24. The MCID for change in walk distance was estimated as 41 m. Patients experiencing an MCID on treatment had worse lung function, lower 6 min walk test distance, higher levels of proinflammatory biomarkers including TIMP-1 and M-CSF, and lower levels of collagen 1a at baseline. Experiencing an MCID during treatment was associated with increases in forced expiratory volume in 1-s [odds ratio (OR) = 1.17, 95% confidence interval (CI) = 1.05–1.33] and increases in blood collagen 1a levels (OR = 1.31, 95%CI 1.06–1.62).

**Conclusions:**

ART and TB treatment are associated with substantial improvements in 6 min walk test distance over time. Achievement of an MCID in the 6 min walk test in this study was associated with more severe disease at baseline and increases in collagen 1a levels and lung function during therapy.

**Supplementary Information:**

The online version contains supplementary material available at 10.1186/s12890-023-02325-7.

## Background

Tuberculosis (TB) remains a major cause of morbidity and mortality worldwide, causing 10 million cases and 1 million deaths globally in 2021 [1]. While a majority of individuals with TB complete treatment, approximately half of TB survivors have objective evidence of impaired pulmonary function after cure [2–4], and a history of TB is associated with reduced long-term survival [3, 5, 6]. When health losses due to TB are quantified as disability-adjusted life-years (DALYs), 47% of the total burden estimate is attributed to post-TB sequelae [5, 6]. With an estimated 155 million TB survivors alive in 2020 [7], it is important to understand factors and mechanisms related to lung function and exercise capacity during TB treatment [4, 8].

Numerous studies evaluating post-TB sequelae have focused on pulmonary function testing [2, 3, 6, 9, 10]. However, TB exerts a negative impact on health that is not entirely captured by assessments of lung function [11–13]. Consistent with this, reduced lifespan among pulmonary TB survivors has been attributed to higher mortality from non-respiratory etiologies including cancer and cardiovascular disease [14]. Global assessments of physical function may complement lung function testing when evaluating TB sequelae. The 6 min walk test, a simple evaluation of sub-maximal exercise capacity that involves a patient walking comfortably for 6 min on a marked course, is one type of test that measures physical function [15]. The distance walked during this time is predictive of mortality in multiple cardiopulmonary diseases and is responsive to changes mediated by interventions [15, 16]. A few studies have documented shorter distances walked both at the time of pulmonary TB diagnosis and after treatment completion [11, 12], which may be due not only to respiratory pathology but also to wasting, physical deconditioning, and overall functional decline. However, among those with TB there are very few data identifying factors associated with the physical function or 6 min walk test distance. This is particularly true in HIV co-infection due to increased inflammation [17–20], which complicates over half of all TB cases in sub-Saharan Africa [1].

Both HIV and TB can cause immune activation and inflammation, which have been associated with worse physical function in various settings [21–24]. For example, higher levels of CRP, IL-6, IL1-RA, and TNFa, as well as higher levels of markers of microbial translocation, have been associated with shorter distances walked and lower functional status among elderly adults [25–27]. Mechanistically, inflammatory cytokines in HIV/TB could be linked indirectly to functional status, by resulting from a more advanced stage of either infection, or directly, by adverse effects on muscle catabolism [8–10]. Patients with HIV/TB are also at risk of the TB immune reconstitution inflammatory syndrome (TB-IRIS), which may acutely raise inflammation [28, 29]. One South African HIV/TB cohort showed immediate reductions in 6 min walk test distances after initiating ART, most dramatically in those with IRIS, suggesting that inflammation mediated by ART-induced immune recovery transiently impaired physical function [17].

Therefore, we evaluated the physical function of patients with HIV/TB co-infection over time after initiation of ART and anti-tubercular therapy using the 6 min walk test. To further understand why patients experience restoration or alleviation of physical health from treatment, we related walk test distance results to markers of inflammation and immune activation which may affect mobility. Yet, biomarker studies have generally not linked the biomarkers to physical function or 6 min walk distances. We selected biomarkers based on their known role in respiratory diseases, including pulmonary TB, and these included matrix metalloproteinases (MMPs), tissue inhibitor of MMP (TIMP)-1, plasminogen activator, vascular endothelial growth factor (VEGF) and collagen 1a, which are associated with lung tissue matrix-remodeling [30], cytokines that contribute to tissue inflammation (IL-6, IL-8, chemokine (C–C) motif-2 (CCL-2), macrophage colony-stimulating factor (M-CSF)) [24], and chemokines and cell adhesion molecules (CXCL-10, CXCL-11, vascular cell adhesion molecule 1 (VCAM-1)) that are involved in migration or trafficking of monocytes, macrophages or leucocytes to sites of infection, including the lung [24, 31, 32].

## Methods

### Study population and procedures

This is a secondary analysis of The Lung Function after TB-IRIS (LIFT-IRIS) study, a prospective cohort study conducted in Gauteng, South Africa. Between July 2016 to March 2018, the LIFT-IRIS study enrolled adults (> 18 years) living with HIV and TB who started anti-TB treatment within 30 days of TB diagnosis and were ART-naïve. Those who have known or suspected resistance to TB treatment, who have received ART medications within the previous 90 days, or who have any conditions (e.g., pregnancy, altered mental status, substance abuse, etc.) affecting or affected by anti-TB treatment or ART were excluded from the LIFT-IRIS study. The baseline visit occurred prior to ART initiation, and patients subsequently had follow-up visits up to 48 weeks after ART initiation [2]. Six-minute walk test distance, lung function, and symptom scores on the chronic obstructive pulmonary disease (COPD) assessment test (CAT) were evaluated at the baseline and at weeks 4, 12, and 24, and biomarkers were measured at baseline, week 4, and week 12, during the time of greatest TB-associated inflammation. (Additional file 2: Figure S1) Patients were treated for HIV and TB consistent with South African National Guidelines [33]. For the current analysis, we limited our study population to those who successfully completed the 6 min walk test at both baseline and week 24 and who did not experience treatment failure.

### Six-minute walk test and minimum clinically important differences

All participants received a detailed explanation of the walk test prior to participation, and were asked to walk back and forth on a 30-m, pre-marked, level pathway in an open space in the research clinic as many times as possible in 6 min using instructions and encouragement in accordance with published guidelines [15]. In order to determine if the change in 6 min walk distance at week 24 represented a meaningful response to treatment, we compared each patient’s change in 6 min walk test distance from baseline to week 24 and divided patients into two groups: minimum clinically important difference (MCID) versus non-MCID. Conceptually, the MCID would be a clinically meaningful change that is correlated with other metrics important to patients' perception of health [34, 35], including symptoms and quality of life (e.g., an anchor-based approach to defining MCID) [36, 37]. Since such validated definitions in TB do not currently exist for this test, we first explored the relationship of CAT scores to walk distance, but they were weakly correlated (r = 0.28; *p* < 0.01), indicating that an anchor-based approach using data available to us was unlikely to be valid [34, 37, 38]. The cutoff to group patients was therefore determined based on a distribution-based estimation by calculating Cohen’s moderate effect size [38], which is half of the standard deviation of changes in 6 min walk test distances from baseline to week 24, as described. Patients who walked further than Cohen’s moderate effect size were classified as experiencing an MCID [34].

### Lung function

Pulmonary function tests were conducted using an EasyOne Pro spirometer (New Diagnostic Designs Medical Technologies, Andover, MA). Forced expiratory volume in 1 s (FEV_1_) and forced vital capacity (FVC) were measured as absolute values and percent-predicted values with standard adjustment for age, height, sex, and race [2].

### Quantitation of plasma markers

Whole blood samples (50 ml) were collected from patients via phlebotomy at the baseline visit (prior to ART initiation), and at 4 and 12 weeks after ART initiation. Plasma samples were separated from the whole blood during the process of Peripheral blood mononuclear cells (PBMC) isolation and cryopreserved at − 80 °C until needed. Custom multi-analyte bead kits purchased from R&D systems, USA were used to determine the concentrations of plasma analytes, focusing on selected biomarkers of tissue remodeling and inflammation. Multiplex analyses were performed as described by the manufacturer and collagen 1a and TIMP metallopeptidase inhibitor 1 (TIMP-1) were determined from plasma samples diluted 50-fold while C–C motif chemokine ligand 2 (CCL-2), C-X-C motif chemokine 11 (CXCL-11), matrix metallopeptidase 1 (MMP-1), vascular cell adhesion molecule 1 (VCAM-1), C-X-C motif chemokine 10 (CXCL-10), macrophage colony-stimulating factor (M-CSF), plasminogen activator, vascular endothelial growth factor (VEGF), IL-8 and IL-6 were measured in plasma diluted twofold. Beads were acquired on a Bio-Plex 200 instrument (Bio-Rad) and the data analyzed using the Bio-Plex manager software (version 6.1, Bio-Rad) capable of generating a five-parameter logistic (5-PL) curve fit. Biomarkers serum levels were measured in pg/L.

### Statistical analyses

We described categorical variables as frequency and percentage and continuous variables as mean and standard deviation. Our main analyses focused on (i) how the odds of experiencing an MCID at week 24 related to baseline characteristics, including lung function and biomarker levels using logistic regression; and (ii) associations between changes in clinical characteristics from baseline to week 4 and from baseline to week 12 using logistic regression.

We conducted secondary analyses using the 6 min walk test distance at week 24 as a continuous outcome using linear regression. Normality in the 6 min walk test distance was confirmed using a QQ plot and Shapiro test. Age and sex were considered as confounders and were included as covariates in the models if associated with the outcome. In addition, we explored relationships between clinical factors and biomarkers and radiographic extent of disease in a subset of 49 patients who completed fluorodeoxyglucose (FDG) positron emission tomography-computed tomography (PET-CT) at baseline. The radiographic extent of the disease was measured as previously described [39].

Lung function and biomarkers were scaled by the number of digits of their standard deviation so that the magnitude of the coefficient would not be determined by the absolute level of biomarkers and that a one-unit change in biomarker levels being compared on the scale is meaningful. All analyses were conducted in R version 3.7.1.

## Results

### Baseline demographic and clinical information

Of 128 patients with a 6 min walk distance at baseline, 89 (70%) completed the test again at week 24 and are included in this analysis. Those included were not meaningfully different from those excluded (Additional file 1: Table S1). The average age of the participants enrolled in the study was 37 years (± 8.1), 42% of whom were female. The average pre-ART CD4 count was 148 cells/mm^3^ (± 133) (Table 1). The mean time between the start date of TB treatment and ART initiation was 30 days (± 18), when 44% of patients were still sputum culture-positive (Table 1). Less than 1% of the study patients reported comorbidities including pre-existing lung diseases which were reflected by the CAT score (mean 7.3, standard deviation 6.3 at baseline, Table 1).

**Table 1:** Characteristics at baseline and during follow-up for adults with HIV and pulmonary TB*

	Baseline (N = 89)	Week 4 (N = 81)	Week 12 (N = 83)	Week 24 (N = 89)
*Demographics*				
Age (year, mean ± standard deviation)	37.2 (± 8.1)	–	–	–
Male sex (n, %)	52 (58%)	–	–	–
Ever smoke (n, %)	32 (36%)	–	–	–
*Clinical characteristics (mean* ± *standard deviation)*				
6MWT^a^ (meters)	393 (± 68.5)	400 (± 55.6)	413 (± 55.4)	430 (± 69.6)
Time between anti-tuberculosis treatment and antiretroviral therapy (days)	30 (± 18)			
Sputum culture positivity^b^ (count (%))	39 (44%)	–	–	–
Body mass index (kg/m^2^)	19.9 (± 3.4)	–	–	–
CD4 count (cells/mm^3^)	148 (± 133)	–	–	–
Plasma HIV RNA level (log_10_ (copies/mm^3^))	5.31 (± 1.1)	–	–	–
FVC (liters)	3.1 (± 0.9)	3.10 (± 0.8)	3.17 (± 0.8)	3.29 (± 0.8)
FVC predicted (%)^a^	83.4 (± 19.0)	84.7 (± 19.1)	86.3 (± 15.2)	88.0 (± 15.5)
FEV_1_ (liters)	2.4 (± 0.7)	2.3 (± 0.6)	2.4 (± 0.7)	2.5 (± 0.6)
FEV_1_ predicted^a^ (%)	76.6 (± 19.4)	75.8 (± 18.5)	78.9 (± 18.5)	80.7 (± 15.2)
COPD assessment test total score^a^	7.3 (± 6.3)	3.8 (± 5.0)	1.5 (± 2.8)	0.7 (± 1.7)
*Biomarkers (mean* ± *standard deviation)*				
Collagen Ia^a^ (pg/mL)	2520 (± 1840)	2740 (± 1780)	5180 (± 2630)	–
TIMP-1^a^ (ng/)	179 (± 106)	185 (± 113)	148 (± 102)	–
CCL-2^a^ (pg/mL)	230 (± 106)	183 (± 75.8)	187 (± 84.7)	–
CXCL-11^a^ (pg/mL)	101 (± 86.8)	66.3 (± 82.8)	56.7 (± 70.5)	–
IL-8 (pg/mL)^a^	13.0 (± 7.38)	12.7 (± 9.27)	8.79 (± 4.34)	–
MMP-1 (pg/mL)^a^	1040 (± 809)	1070 (± 745)	843 (± 605)	–
VCAM-1 (ng/mL)^a^	2240 (± 1320)	1810 (± 1370)	1210 (± 707)	–
CXCL-10 (pg/mL)^a^	534 (± 637)	398 (± 512)	206 (± 160)	–
IL-6 (pg/mL)^a^	7.31 (± 10.9)	10.1 (± 29.2)	3.48 (± 7.39)	–
M-CSF (pg/mL)^a^	187 (± 128)	164 (± 129)	113 (± 84.6)	–
Plasminogen activator (pg/mL)^a^	930 (± 318)	885 (± 300)	751 (± 233)	–
VEGF (pg/mL)	17.0 (± 9.49)	15.9 (± 10.6)	14.4 (± 10.1)	–

Data are presented as n (%)

*6MWT* six minute walk time, *BMI* body mass index, *VL* vial load, *FVC* forced vital capacity, *FEV*_*1*_ forced expiratory volume in 1 s, *COPD* chronic obstructive pulmonary disease

*Missing—11% in ever smoke, BMI, CD4, viral load, FVC, FVC predicted, FEV_1_, and FEV_1_ predicted; and 5% in biomarkers

^a^Significant time effect based on within-subject analysis of variance at alpha = 0.01

^b^Study recruitment at baseline was within 30 days from anti-TB treatment initiation. Some samples may already be “culture negative”

At baseline, older age was associated with shorter 6 min walk test distance measured as a continuous variable, whereas male sex and higher FEV_1_% predicted were associated with a longer distance walked (Additional file 1: Table S2). Higher levels of CXCL-11 and M-CSF were significantly associated with a shorter 6 min walk test distance at baseline after adjusting for age and sex (Additional file 1: Table S2). Point estimates for associations between baseline 6 min walk test distance and baseline biomarker levels were all negative except for collagen 1a [2.39 (95%CI − 4.56, 9.34), Additional file 1: Table S2].

### Changes in 6 min walk test, lung function, and biomarkers over time

After ART initiation, the mean 6 min walk test distance increased from 393 (± 68.5) meters at baseline to 430 (± 69.6) meters at week 24, representing an approximate 9% improvement over time. Similarly, lung function (FEV_1_ and FVC) improved by approximately 4%, and CAT scores rapidly decreased (Table 1, Additional file 2: Fig. S1). Of the 12 plasma biomarkers measured, CCL-2, CXCL-11, IL-8, VCAM-1, CXCL10, M-CSF, plasminogen activating factor and VEGF declined following treatment, whereas MMP-1, TIMP-1, and IL-6 increased at week 4 but declined by week 12 (Table 1 and Additional file 2: Fig. S1). Only collagen 1a increased throughout follow-up, more than doubling between baseline and week 12 (Table 1 and Additional file 2: Fig. S2).

### Minimum clinically important difference (MCID) in 6 min walk test

Using a distribution method based on Cohen’s moderate effect size, the cutoff for MCID in 6 min walk test distance during 24 weeks of treatment was estimated as 41 m; 43 (48%) patients had an increase in the 6 min walk test distance equal to this distance or greater (Table 2). Neither age nor sex were confounders in this analysis and therefore unadjusted associations are presented. Patients who experienced an MCID from baseline to week 24 were more likely to be past smokers and tended to present with lower CD4 counts, indicating more advanced HIV disease (Table 2). In addition, those with an MCID tended to have higher CAT scores, lower FEV_1_ and FVC, and a shorter 6 min walk test distance at baseline (Table 2). Higher pre-ART levels of TIMP-1 and MCSF, and lower levels of collagen 1a, were also associated with an increased odds of having an MCID from baseline to week 24 (Table 2).

**Table 2:** Factors associated with a minimum clinically important difference in 6 min walk test according to a distribution-based definition. Distribution-based approach following Cohen’s moderate effect size, 41 m in this study cohort

	Minimum clinical important difference (N = 43)	No minimum difference (N = 46)	Scale factor	Unadjusted odds ratio of MCID
	Baseline	Baseline		
	(N = 43)	(N = 46)		
*Demographics*				
Age (year, mean ± standard deviation)	37.3 (± 7.6)	37.1 (± 8.6)		1.00 (0.95, 1.06)
Male sex (n, %)	24 (56%)	28 (61%)		0.81 (0.35, 1.89)
Ever smoke (n, %)	19 (44%)	13 (28%)		2.58 (1.04, 6.63)
*Clinical characteristics (mean* ± *standard deviation)*				
Six-minute walk distance (meters)^a^	357 (± 60)	427 (± 59)	10 m	0.83 (0.76, 0.90)
Sputum culture positivity* (count (%))	16 (37%)	23 (50%)		0.59 (0.25, 1.37)
Time between anti-tuberculosis treatment and antiretroviral therapy (days)	33 (± 20)	26 (± 16)		1.02 (1.00, 1.05)
Body mass index (kg/m2)	19.3 (± 2.4)	20.4 (± 4.1)		0.90 (0.76, 1.03)
CD4 count (cells/mm^3^)	127 (± 135)	165 (± 130)	10 cells/mm3	0.98 (0.94, 1.01)
Plasma HIV RNA level (_log10_ (copies/mm^3^))	5.4 (± 1.0)	5.2 (± 1.1)		1.14 (0.75, 1.77)
Forced vital capacity (FVC, liters)	3.0 (± 0.8)	3.2 (± 0.9)	100 mL	0.97 (0.92, 1.02)
FVC predicted (%)^a^	81.2 (± 19.6)	85.7 (± 18.3)	5%	0.94 (0.83, 1.06)
Forced expiratory volume in 1 s (FEV_1_, liters)	2.2 (± 0.7)	2.5 (± 0.7)	100 mL	0.94 (0.87, 1.00)
FEV_1_ predicted (%)	72.7 (± 19.5)	80.5 (± 18.7)	5%	0.90 (0.79, 1.01)
COPD** Assessment test total score	8.0 (± 6.7)	6.6 (± 6.0)		1.04 (0.97, 1.11)
*Biomarkers (mean* ± *standard deviation)*				
Collagen Ia (pg/L)^a^	2080 (± 1170)	2930 (± 2230)	1000 pg/L	0.72 (0.49, 0.96)
TIMP1 (pg/L)^a^	208,000 (± 110,000)	152,000 (± 95,500)	10000 pg/L	1.05 (1.01, 1.10)
CCL2 (pg/L)	224 (± 109)	236 (± 105)	100 pg/L	0.89 (0.59, 1.34)
CXCL11 (pg/L)	109 (± 88.1)	94.1 (± 86.0)	10 pg/L	1.02 (0.97, 1.07)
IL8 (pg/L)	13.0 (± 6.94)	13.0 (± 7.85)	1 pg/L	1.00 (0.94, 1.06)
MMP1 (pg/L)	990 (± 595)	1090 (± 972)	100 pg/L	0.98 (0.93, 1.04)
VCAM1 (pg/L)	2,450,000 (± 1,550,000)	2,040,000 (± 1,030,000)	1000000 pg/L	1.28 (0.92, 1.85)
CXCL10 (pg/L)	506 (± 339)	561 (± 828)	100 pg/L	0.99 (0.91, 1.06)
IL6 (pg/L)	6.3 (± 9.4)	8.3 (± 12.3)	10 pg/L	0.83 (0.49, 1.25)
M-CSF (pg/L)^a^	213 (± 130)	162 (± 124)	10 pg/L	1.03 (1.00, 1.07)
Plasminogen activator (pg/L)	955 (± 335)	907 (± 304)	100 pg/L	1.05 (0.92, 1.21)
VEGF (pg/L)	17.1 (± 9.9)	16.9 (± 9.2)	1 pg/L	1.00 (0.96, 1.05)

Missing—11% in ever smoke, BMI, CD4, viral load, FVC, FVC pred, FEV1, and FEV1pred; 4% in time to ART; and 5% in biomarkers

*Study recruitment at baseline was within 30 days from anti-TB treatment initiation, and hence the culture results may already be conversed to negative

**COPD, Chronic Obstructive Pulmonary Disease

^a^Statistically significant difference in mean values at baseline at alpha = 0.05

### Factors associated with experiencing a MCID in the 6 min walk test

**Fig. 1:**
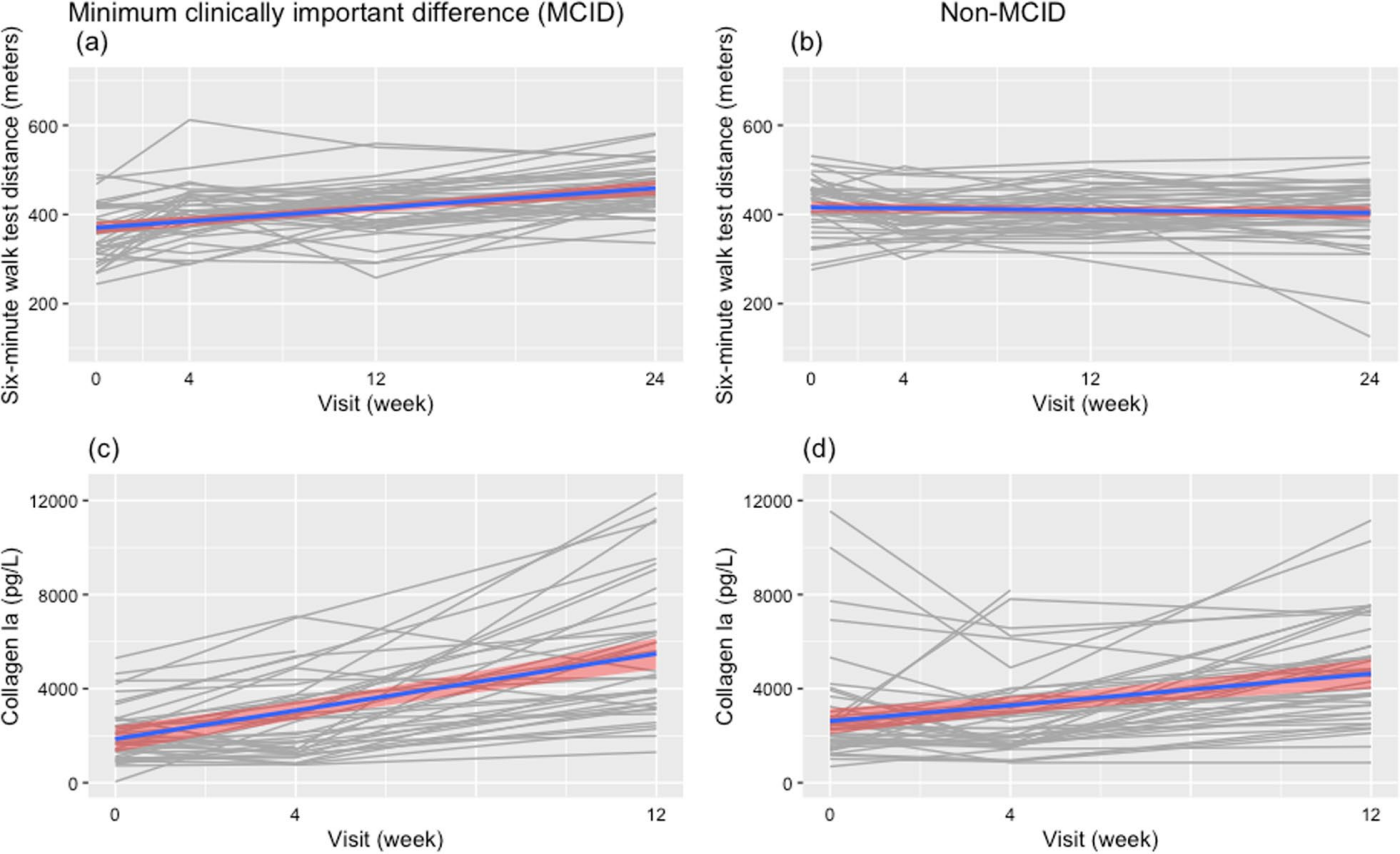
Mean change in 6MWT distance and collagen 1a over time in patients with and without MCID. Changes over time in 6MWT distance (meters) and plasma levels of collagen 1a (pg/L) in patients with MCID (n = 43; **a** and **c**) and without MCID (n = 46; **b** and **c**). Six-minute walk test distance results are presented for participants followed up until week 24. Plasma collagen 1a levels measured at the week 12 visit are shown. Grey lines represent the changes for each individual. The blue line represents the mean of changes and the red band the 95% confidence intervals

The odds of experiencing an MCID in 6 min walk distance related to changes in collagen 1a levels, and this differed compared to changes in other plasma biomarkers. (Fig. 1 and Additional file 1: Table S3). An increased odds of achieving an MCID from baseline to week 24 was significantly associated with greater increases in FEV_1_ percent predicted [odds ratio (OR) = 1.20, 95% confidence Interval (95%CI) 1.03, 1.43, *p-*value = 0.04] and FVC percent predicted (OR = 1.22, 95%CI 1.01, 1.52, *p-*value = 0.05) between baseline and week 12 (Table 3). Notably, increasing levels of collagen 1a from baseline to both week 4 and week 12 (OR = 1.31, 95%CI 1.08, 1.62, *p*-value = 0.02), and increasing levels of CXCL-10 from baseline to week 4 (OR = 1.08, 95%CI 1.00, 1.22, *p*-value = 0.05), were significantly associated with an increased adjusted odds of experiencing an MCID in the 6 min walk test distance (Table 3). When the 6 min walk test distance at week 24 was modeled as a continuous variable, increases in collagen 1a levels over time remained associated with greater distance walked (Additional file 1: Table S4). In addition, higher levels of TIMP-1 at baseline, and greater decreases in TIMP-1 levels from baseline to week 12, were associated with greater distance walked at week 24. Similarly, a decrease in VCAM-1 over time was also associated with greater distance walked (Additional file 1: Table S4).

**Table 3:** Odds (and 95% confidence intervals) of experiencing the minimum clinically important difference in 6 min walk distance from baseline to week 24 associated with lung function and biomarker values at baseline, changes in values from baseline to week 4, and changes in values from baseline to week 12

	Changes from baseline to week 4 (N = 77), OR (95% CI)	Changes from baseline to week 12 (N = 78), OR (95% CI)
Forced vital capacity (FVC, 100 ml)*	1.03 (0.94, 1.13)	1.11 (1.00, 1.25)
FVC predicted (%)*	1.08 (0.92, 1.28)	1.22 (1.01, 1.52)
Forced expiratory volume in 1 s (FEV_1_, 100 ml)*	1.10 (0.99, 1.24)	1.17 (1.05, 1.33)
FEV_1_ predicted (%)*	1.13 (0.98, 1.34)	1.20 (1.03, 1.43)
COPD** assessment test total score	0.97 (0.91, 1.04)	0.94 (0.87, 1.01)
Collagen Ia (1000 pg/L)	1.32 (0.99, 1.83)	1.31 (1.08, 1.62)
TIMP1 (100000 pg/L)	1.00 (0.93, 1.07)	0.97 (0.91, 1.03)
CCL2 (100 pg/L)	1.32 (0.75, 2.40)	1.41 (0.88, 2.35)
CXCL11 (10 pg/L)	1.01 (0.95, 1.08)	1.01 (0.95, 1.07)
IL8 (pg/L)	1.02 (0.97, 1.07)	1.02 (0.94, 1.11)
MMP-1 (100 pg/L)	1.02 (0.96, 1.10)	1.02 (0.96, 1.08)
VCAM1 (1000000 pg/L)	0.75 (0.50, 1.06)	0.69 (0.43, 1.06)
CXCL10 (100 pg/L)	1.08 (1.00, 1.22)	1.02 (0.95, 1.12)
IL6 (10 pg/L)	1.12 (0.93, 1.68)	1.13 (0.64, 2.15)
M-CSF (100 pg/L)	1.03 (0.98, 1.08)	0.99 (0.94, 1.03)
Plasminogen activator (100 pg/L)	0.88 (0.72, 1.05)	1.01 (0.86, 1.19)
VEGF (pg/L)	1.01 (0.97, 1.06)	1.02 (0.97, 1.07)

*n = 79 at baseline, n = 68 at changes at week4, and n = 63 at changes at week12

**COPD, Chronic obstructive pulmonary disease

### Supplementary analyses

We have shown that higher levels of circulating collagen 1a appear to be associated with improved walk distance during HIV/TB treatment; however, physical factors affecting the 6 min walk test could be related to both pulmonary and extrapulmonary inflammation. We have previously shown that greater lung involvement on FDG PET-CT is associated with worse lung function. We therefore explored whether the levels of biomarkers in blood were associated with radiographic lung involvement using patients in the PET-CT sub-study. (Additional file 1: Table S5 and Additional file 2: Fig. S3) These analyses revealed that higher collagen 1a, CCL2, and VCAM-1 levels were associated with less lung involvement, and that higher levels of IL-6, MMP-1, and VEGF were associated with worse lung involvement at baseline.

## Discussion

In this cohort study from a high TB and HIV burden setting in sub-Saharan Africa, we prospectively evaluated clinical factors and biomarkers associated with the 6 min walk test distance in adults being treated for HIV/TB. Our primary clinical finding is that substantial increases in this test occur within 24 weeks of HIV and TB treatment, are associated with improvements in lung function, and are more likely to be larger in those who present with more severe disease. Our primary biomarker findings are that both baseline levels and changes in levels of markers of inflammation and tissue remodeling are associated with the 6 min walk test distance at baseline and over time. Notably, collagen 1a is unique among the biomarkers assessed in that increasing levels during the first 12 weeks of treatment are associated with greater gains in the 6 min walk test distance from baseline to week 24.

Previous studies showed that a history of pulmonary TB has been associated with substantially reduced 6 min walk test distances, highlighting that disease sequelae can impact on overall physical function [11–13]. However, relatively few studies have evaluated changes in this parameter during therapy [40], and no studies to our knowledge have attempted to determine clinically meaningful changes in this outcome over the duration of treatment in patients with pulmonary TB. With the use of a distribution-based method, we identified two groups that differed in baseline characteristics including CD4 counts, TIMP-1 and IL-6 levels, and pre-ART 6 min walk distance. Patients with worse disease at baseline (e.g. higher CAT scores, lower CD4 counts, and lower lung function measures) were more likely to experience a meaningful change in 6 min walk test distance during treatment, indicating that those with the greatest baseline impairment stand to have the greatest functional benefit from therapy, giving hope to individuals with advanced disease.

Determining a clinically meaningful difference in this test in TB patients can be useful for future study planning and for sample size calculations for trials assessing treatments or strategies designed to improve physical function during anti-tubercular therapy [17]. Our study was a secondary analysis using the existing cohort data, but further research may specifically conduct a sample size calculation for the comparisons of interest and may inform if the meaningful change as determined here is generalizable. Our study population walked approximately 100–200 m shorter at baseline compared to other TB studies [11, 12, 17], which suggests that meaningful differences measured in meters may be setting-specific, varying according to cohort functional status, patient instructions during the test, or possibly characteristics of the track and environment. However, the relative degree of improvement in our study is comparable to data from Stek et al. [11, 12, 17] and other reports, suggesting that a relative change expressed as a percent of baseline, as used in other respiratory diseases, may be more useful.

Analyses relating biomarker levels to 6 min walk test distance focused on factors involved broadly in inflammation and tissue remodeling, and are meant to provide insights into how the resolution of inflammation and activation of tissue repair mechanisms may relate to recovery from illness as defined by a functional, patient-reported outcome. At baseline, higher levels of TIMP-1 and MCSF were associated with both increased odds of achieving an MCID in the walk test at week 24 as well as with greater distance walked at week 24. These results are consistent with sicker patients with higher levels of inflammation experiencing more dramatic increases in walk distance in response to therapy [17]. MMPs which are inhibited by TIMPs, degrade lung collagen and have been associated with lung immunopathology, disease severity, and treatment outcomes in TB [31, 41, 42]. Previous studies, primarily in chronic heart failure patients, have also found inverse associations between TIMP-1 levels and functional outcomes, including impaired exercise oxygen consumption, increased cardiovascular endpoints, and shorter 6 min walk test distance [43, 44]. While TIMP-1 levels in this cohort were not associated with radiographic metrics of disease severity, MMP-1 levels were strongly associated with worse lung involvement in the subset with FDG PET-CTs. Delineation of precise pathways relating circulating biomarker levels to specific pathophysiology in this study is complicated by the holistic outcome measure evaluated, but these data support a relationship between extracellular matrix remodeling and a clinically relevant functional outcome as assessed by the 6 min walk test in TB.

This study revealed relatively robust associations between levels of collagen 1a and 6 min walk test distance prior to ART initiation and during treatment. Specifically, lower pre-treatment levels were associated with greater odds of achieving an MCID at week 24, and greater increases during treatment were associated with greater increases from baseline to week 24. These results contrast with results from other pulmonary diseases including idiopathic pulmonary fibrosis and COPD, where higher levels have most often been associated with worse disease [45, 46]. Collagen 1a is expressed by cells in the respiratory tract and various other tissues including bone and cartilage, and therefore it is possible that higher levels reflect greater muscle mass or other extrapulmonary characteristics that could manifest as greater sub-maximal exercise capacity [47]. Furthermore, it is possible that higher activity levels lead to higher collagen 1a levels in the blood, as has been demonstrated in medium and long-term exercise physiology studies [48]. Given advanced HIV and TB are both associated with wasting and a generalized catabolic state that is reversed by therapy, we suspect that some component of the association between collagen 1a levels and improved 6 min walk test distance at week 24 is attributable to the extent of recovery from a greater injury prior to ART and extrapulmonary changes during treatment.

In addition, circulating collagen 1a levels in these patients were related to pulmonary manifestations of the disease. Our exploratory analyses among 49 patients who had FDG PET-CT imaging at baseline showed that higher collagen 1a levels were consistently correlated with lower radiographic involvement and therefore lower levels of lung enhancement on FDG PET and lower structural lung involvement on CT at baseline. (Additional file 1: Table S5 and Additional file 2: Fig. S2) Type I and III fibrillar collagens are key structural components of the lung extracellular matrix and a few enzymes, namely MMP-1, MMP-8, MMP-13, MMP-14, and cathepsin K (CTSK), are able to cleave collagen [49]. Mechanistically, patients with the greatest lung involvement and therefore the greatest MMP-mediated collagen degradation would have the least intact collagen 1a detectable in blood. Several studies on TB have identified associated higher airway MMP levels and higher blood levels of biomarkers of the lung extracellular matrix, including desmosine and PIIINP [47], with more severe TB disease radiographically. Our study suggests collagen 1a may have an opposite relationship and can be a biomarker of relative health, although this requires future study.

Limitations include a high rate of loss to follow up (30%) from baseline to week 24. Although we found no differences in the characteristics at baseline between patients who completed the study versus those lost-to-follow-up, non-participation remains a potential source of bias as unmeasured comorbidities may have affected the follow-up rate. Second, we did not track CD4 counts or body mass index changes during the course of ART which may have been associated with the 6 min walk test distance at week 24. Third, we do not have levels of plasma markers beyond week 12. Future studies that include measuring biomarker levels after the known intense inflammatory period may give additional clues to mechanisms related to post-TB sequelae. Fourth, measurement bias in the 6 min walk test may be introduced because of patient misunderstanding of the test instructions. However, we followed the standard guidelines for conducting a 6 min walk test as recommended by the American Thoracic Society. Fifth, our target population is TB/HIV co-infected adult patients, and sub-maximal exercise capacity in TB patients without HIV infection (or HIV-infected patients without TB disease) may have different relationships than those discovered here. Further studies using alternative control groups may supplement our study findings beyond the TB/HIV co-infected patients. Finally, determining an MCID is challenging since any cutoff may be setting-specific and may depend on a particular cohorts’ characteristics. Despite large differences in the absolute 6 min walk distances from other populations, we are reassured by the fact that the relative degree of improvement we measured is compatible to that of other studies (12% from our study vs. 8–9% in other studies), implying compatible MCID classifications in TB cohorts [17].

In summary, we identified an MCID in the 6 min walk test distance among HIV/TB patients receiving ART and anti-TB therapy using a distribution-based method. We found that more symptomatic patients presenting with lower lung function, shorter 6 min walk test distances, and higher pre-ART levels of proinflammatory biomarkers including TIMP-1 and M-CSF were more likely to achieve an MCID in the 6 min walk test distance during therapy. Furthermore, improvements in lung function and decreases in certain biomarkers, including TIMP-1 and VCAM-1, during therapy were also associated with greater distances walked at follow-up. In addition, among the biomarkers we assessed, we found a unique relationship between collagen 1a levels and 6 min walk test distances. Specifically, lower collagen 1a levels at baseline, and greater increases in collagen 1a levels during treatment, were associated with greater odds of achieving an MCID in the 6 min walk test over time. Taken together, these findings highlight the beneficial effects of treating HIV and TB on physical function and suggest distinct dynamics of inflammatory and certain tissue repair processes. Furthermore, the 6 min walk test provides some insight into the physical capacity of patients beyond lung function, and therefore may serve as a complementary measure to follow up post-TB sequelae.

## Supplementary Information

**Additional file 1**. Supplementary Tables.

**Additional file 2**. Supplementary Figures.

## Data Availability

The datasets used and/or analyzed during the current study are available from the corresponding author on reasonable request.
