# Supplementary material for: Lung function and collagen 1a levels are associated with changes in 6 min walk test distance during treatment of TB among HIV-infected adults: a prospective cohort study
Source: BMC Pulm Med. 2023 Feb 3;23:53. doi: 10.1186/s12890-023-02325-7 (PMC9896708; doi:10.1186/s12890-023-02325-7)
Supplement: Supplementary file 1 — Additional file 1. Supplementary Tables. [file 12890_2023_2325_MOESM1_ESM.docx]

**Supplementary Tables**

**Table S1. Patients lost to follow up at week 24 compared to the study patients**

|  | **Patients lost to follow up at week 24**  **(N=39)** | **Study patients (N=89)** |
| --- | --- | --- |
| **Demographics (count (%))** |  |  |
| **Age (year, mean ± standard deviation)** | **37.0 (± 8.56)** | **37.2 (± 8.1)** |
| **Male sex** | **22 (56.4%)** | **52 (58%)** |
| **Ever smoke** | **16 (41.0%)** | **32 (36%)** |
| **Clinical characteristics (mean ± standard deviation)** |  |  |
| **Six-minute walk distance (meter)** | **378 (± 92.4)** | **393 (± 68.5)** |
| **Sputum culture positivity* (count (%))** | **20 (51.3%)** | **39 (44%)** |
| **Body mass index (kg/m2)** | **20.0 (± 3.45)** | **19.9 (± 3.4)** |
| **CD4 count (cells/mm3)** | **122 (± 116)** | **148 (± 133)** |
| **Plasma HIV RNA level (_log10_ (copies/mm3))** | **5.58 (± 1.33)** | **5.31 (± 1.1)** |
| **Forced vital capacity (FVC, liters)** | **3.05 (± 0.841)** | **3.1 (± 0.9)** |
| **FVC predicted (%)** | **82.3 (± 17.7)** | **83.4 (± 19.0)** |
| **Forced expiratory volume in 1 second (FEV_1_, liters)** | **2.37 (± 0.754)** | **2.4 (± 0.7)** |
| **FEV_1_ predicted (%)** | **75.8 (± 18.7)** | **76.6 (± 19.4)** |
| **COPD* Assessment Test total score** | **8.26 (± 6.40)** | **7.3 (± 6.3)** |
| **Biomarkers (mean ± standard deviation)** |  |  |
| **Collagen Ia (pg/L)** | **2950 (± 1600)** | **2520 (± 1840)** |
| **TIMP1 (pg/L)** | **166000 (± 111000)** | **179000 (± 106000)** |
| **CCL2 (pg/L)** | **233 (± 104)** | **230 (± 106)** |
| **CXCL11 (pg/L)** | **113 (± 84.4)** | **101 (± 86.8)** |
| **IL8 (pg/L)** | **14.0 (± 8.03)** | **13.0 (± 7.38)** |
| **MMP1 (pg/L)** | **1430 (± 1270)** | **1040 (± 809)** |
| **VCAM1 (pg/L)** | **2200000 (± 1460000)** | **2240000 (± 1320000)** |
| **CXCL10 (pg/L)** | **502 (± 525)** | **534 (± 637)** |
| **IL6 (pg/L)** | **7.73 (± 6.89)** | **7.31 (± 10.9)** |
| **MCSF (pg/L)** | **179 (± 89.9)** | **187 (± 128)** |
| **Plasminogen activator (pg/L)** | **950 (± 380)** | **930 (± 318)** |
| **VEGF (pg/L)** | **16.8 (± 8.97)** | **17.0 (± 9.49)** |

* COPD, Chronic Obstructive Pulmonary Disease

None of the variables were significantly different between two group

**Table S2. Association between baseline clinical and biomarker factors and six-minute walk test distance in meters**

|  | **Unadjusted model (95% confidence interval)** | **Adjusted for age and sex (95% confidence interval)** |
| --- | --- | --- |
| **Age (year, mean ± standard deviation)** | -1.87 (-3.63, -0.12) | -2.20 (-3.81, -0.59) |
| **Male sex** | 34.4 (5.91, 63.0) | 56.49 (29.8, 83.2) |
| **Body mass index (kg/m2)** | 1.70 (-2.85, 6.25) | 0.45 (-3.67, 4.57) |
| **CD4 count (cells/mm3)** | -1.21 (-13.0, 10.5) | 2.22 (-8.49, 12.9) |
| **Plasma HIV RNA level (log10 (copies/mm3))** | 6.43 (-7.85, 20.7) | 6.51 (-5.07, 18.1) |
| **Forced vital capacity (FVC, liters)** | 2.30 (0.62, 3.97) | 1.38 (-0.70, 3.45) |
| **FVC predicted (%)** | 2.10 (-1.91, 6.11) | 2.69 (-1.14, 6.52) |
| **Forced expiratory volume in 1 second (FEV_1_, liters)** | 3.43 (1.41, 5.45) | 2.06 (-0.18, 4.30) |
| **FEV1 predicted (%)** | 3.51 (-0.36, 7.39) | 3.86 (0.25, 7.46) |
| **COPD* Assessment Test total score** | -2.01 (-4.28, 0.25) | -1.89 (-3.91, 0.12) |
| **Collagen Ia (1000pg/L)** | 6.28 (-1.79, 14.3) | 2.39 (-4.56, 9.34) |
| **TIMP1 (100000pg/L)** | 0.17 (-1.25, 1.59) | -0.66 (-1.80, 0.47) |
| **CCL2 (100pg/L)** | 5.98 (-8.10, 20.1) | -4.52 (-16.4, 7.40) |
| **CXCL11 (10pg/L)** | -2.09 (-3.76, -0.42) | -1.91 (-3.29, -0.53) |
| **IL8 (pg/L)** | 0.49 (-1.54, 2.53) | -0.01 (-1.63, 1.62) |
| **MMP1 (100pg/L)** | -0.64 (-2.49, 1.22) | -0.60 (-1.84, 0.64) |
| **VCAM1 (1000000pg/L)** | 1.24 (-10.2, 12.7) | -3.72 (-12.7, 5.26) |
| **CXCL10 (100pg/L)** | 0.91 (-1.44, 3.26) | -0.18 (-2.20, 1.84) |
| **IL6 (10pg/L)** | -11.9 (-25.4, 1.64) | -8.71 (-21.4, 3.99) |
| **MCSF (100pg/L)** | -8.73 (-20.3, 2.83) | -11.83 (-22.0, -1.71) |
| **Plasminogen Activator (100pg/L)** | 0.05 (-4.67, 4.78) | -2.28 (-5.89, 1.32) |
| **VEGF (pg/L)** | -0.47 (-2.05, 1.11) | -0.47 (-1.79, 0.84) |

* COPD, Chronic Obstructive Pulmonary Disease

**Table S3. Differences in baseline and follow-up characteristics stratified by achieving a minimum clinically important difference in six-minute walk test according to a distribution-based definition**

|  | **Minimum clinical important difference (N=43)** | | | | **No minimum difference (N=46)** | | | |
| --- | --- | --- | --- | --- | --- | --- | --- | --- |
|  | **Baseline** | **Week4** | **Week12** | **Week 24** | **Baseline** | **Week4** | **Week12** | **Week 24** |
|  | (N=43) | (N=38) | (N=39) | (N=43) | (N=46) | (N=43) | (N=44) | (N=46) |
| **Demographics (count (%))** |  |  |  |  |  |  |  |  |
| **Age (year, mean ± standard deviation)** | 37.3 (± 7.6) | - | - | - | 37.1 (± 8.6) | - | - | - |
| **Male sex** | 24 (56%) | - | - | - | 28 (61%) | - | - | - |
| **Ever smoke** | 19 (44%) | - | - | - | 13 (28%) | - | - | - |
| **Clinical characteristics (mean ± standard deviation)** | | | | | | | | |
| **Six-minute walk distance (meters)^1,2^** | 357 (± 60) | 405 (± 63) | 413 (± 62) | 457 (± 55) | 427 (± 59) | 396 (± 48) | 413 (± 50) | 405 (± 73) |
| **Sputum culture positivity* (count (%))** | 16 (37%) | - | - | - | 23 (50%) | - | - | - |
| **Time between anti-tuberculosis treatment and antiretrotherapy (days)** | 33 (± 20) |  |  |  | 26 (± 16) |  |  |  |
| **Body mass index (kg/m2)** | 19.3 (± 2.4) | - | - | - | 20.4 (± 4.1) | - | - | - |
| **CD4 count (cells/mm3)** | 127 (± 135) | - | - | - | 165 (± 130) | - | - | - |
| **Plasma HIV RNA level (_log10_ (copies/mm3))** | 5.4 (± 1.0) | - | - | - | 5.2 (± 1.1) | - | - | - |
| **Forced vital capacity (FVC, liters)** | 3.0 (± 0.8) | 3.1 (± 0.7) | 3.2 (± 0.8) | 3.2 (± 0.8) | 3.2 (± 0.9) | 3.1 (± 0.9) | 3.1 (± 0.8) | 3.4 (± 0.8) |
| **FVC predicted (%)^1^** | 81.2 (± 19.6) | 85.7 (± 20.6) | 88.9 (± 13.6) | 86.4 (± 13.9) | 85.7 (± 18.3) | 83.9 (± 18.0) | 84.2 (± 16.2) | 89.2 (± 16.6) |
| **Forced expiratory volume in 1 second (FEV_1_, liters)** | 2.2 (± 0.7) | 2.3 (± 0.6) | 2.5 (± 0.7) | 2.4 (± 0.6) | 2.5 (± 0.7) | 2.4 (± 0.7) | 2.4 (± 0.7) | 2.6 (± 0.7) |
| **FEV_1_ predicted (%)** | 72.7 (± 19.5) | 76.0 (± 19.1) | 82.4 (± 16.4) | 78.3 (± 14.3) | 80.5 (± 18.7) | 75.6 (± 18.2) | 76.2 (± 19.8) | 82.6 (± 15.8) |
| **COPD** Assessment Test total score** | 8.0 (± 6.7) | 3.4 (± 4.5) | 1.0 (± 1.4) | 0.3 (± 1.6) | 6.6 (± 6.0) | 4.2 (± 5.4) | 1.9 (± 3.5) | 0.9 (± 1.7) |
| **Biomarkers (mean ± standard deviation)** | | | | | | | | |
| **Collagen Ia (pg/L)^1,2^** | 2080 (± 1170) | 2700 (± 1800) | 5610 (± 2970) | - | 2930 (± 2230) | 2780 (± 1780) | 4800 (± 2260) | - |
| **TIMP1 (pg/L)^1^** | 208000 (± 110000) | 215000 (± 110000) | 166000 (± 104000) | - | 152000 (± 95500) | 159000 (± 110000) | 132000 (± 98300) | - |
| **CCL2 (pg/L)** | 224 (± 109) | 182 (± 79.2) | 189 (± 86.7) | - | 236 (± 105) | 184 (± 73.7) | 185 (± 83.9) | - |
| **CXCL11 (pg/L)** | 109 (± 88.1) | 77.8 (± 102) | 64.2 (± 72.8) | - | 94.1 (± 86.0) | 56.1 (± 60.8) | 49.8 (± 68.6) | - |
| **IL8 (pg/L)** | 13.0 (± 6.94) | 13.2 (± 10.2) | 8.79 (± 4.3) | - | 13.0 (± 7.85) | 12.2 (± 8.50) | 8.80 (± 4.44) | - |
| **MMP1 (pg/L)** | 990 (± 595) | 1050 (± 695) | 802 (± 646) | - | 1090 (± 972) | 1090 (± 795) | 880 (± 570) | - |
| **VCAM1 (pg/L)** | 2450000 (± 1550000) | 1780000 (± 1180000) | 1130000 (± 592000) | - | 2040000 (± 1030000) | 1840000 (± 1540000) | 1270000 (± 798000) | - |
| **CXCL10 (pg/L)** | 506 (± 339) | 516 (± 679) | 218 (± 146) | - | 561 (± 828) | 295 (± 269) | 194 (± 173) | - |
| **IL6 (pg/L)** | 6.3 (± 9.4) | 12.6 (± 41.1) | 2.7 (± 5.2) | - | 8.3 (± 12.3) | 7.9 (± 11.5) | 4.2 (± 8.9) | - |
| **MCSF (pg/L)^1^** | 213 (± 130) | 208 (± 132) | 134 (± 98.1) | - | 162 (± 124) | 125 (± 114) | 95.3 (± 66.5) | - |
| **Plasminogen Activator (pg/L)** | 955 (± 335) | 870 (± 316) | 760 (± 247) | - | 907 (± 304) | 897 (± 287) | 742 (± 222) | - |
| **VEGF (pg/L)** | 17.1 (± 9.9) | 16.5 (± 11.9) | 15.7 (± 12.1) | - | 16.9 (± 9.2) | 15.4 (± 9.3) | 13.2 (± 7.9) | - |

1 Statistically significant difference in mean values at baseline at alpha = 0.05

2 Statistically significant difference in repeated measure analysis of variance (ANOVA) between two groups (in other words, the interaction term of MCID group and time was significant in within-subject tests)

* Missing - 11% in ever smoke, BMI, CD4, viral load, FVC, FVC pred, FEV1, and FEV1pred; and 5% in biomarkers

** COPD, Chronic Obstructive Pulmonary Disease

**Table S4. Six-minute walk distance in meters (95% confidence intervals) at week 24 associated with lung function and biomarker values at baseline, changes in values from baseline to week 4, and changes in values from baseline to week 12, after adjusting for baseline six-minute walk distance**

|  | **Baseline value (N=85)** | **Changes from baseline to week 4 (N=77)** | **Changes from baseline to week 12 (N=78)** |
| --- | --- | --- | --- |
| **Forced vital capacity (FVC, liters)*** | 0.31 (-1.55, 2.17) | -0.36 (-3.53, 2.81) | 2.03 (-1.46, 5.51) |
| **FVC predicted (%)*** | -0.47 (-4.59, 3.65) | -0.92 (-6.38, 4.55) | 3.19 (-2.82, 9.20) |
| **Forced expiratory volume in 1 second (FEV_1_, liters)*** | 0.27 (-2.08, 2.62) | 0.13 (-3.13, 3.39) | 0.43 (-2.86, 3.71) |
| **FEV_1_ predicted (%)*** | -0.37 (-4.46, 3.72) | -0.21 (-5.11, 4.70) | 0.44 (-4.33, 5.21) |
| **COPD** Assessment Test total score** | 0.60 (-1.69, 2.89) | -0.47 (-2.69, 1.75) | -1.18 (-3.41, 1.04) |
| **Collagen Ia (1000pg/L)** | -6.86 (-14.9, 1.22) | 9.51 (0.39, 18.6) | 5.22 (-0.41, 10.9) |
| **TIMP1 (100000pg/L)** | 2.32 (1.01, 3.63) | -0.80 (-3.31, 1.70) | -1.99 (-3.88, -0.09) |
| **CCL2 (100pg/L)** | 1.55 (-12.5, 15.6) | -1.30 (-21.2, 18.6) | -2.98 (-18.9, 13.0) |
| **CXCL11 (10pg/L)** | 0.92 (-0.85, 2.69) | -0.36 (-2.60, 1.88) | -0.50 (-2.38, 1.37) |
| **IL8 (pg/L)** | 1.28 (-0.73, 3.28) | -0.14 (-1.80, 1.52) | -2.49 (-5.19, 0.20) |
| **MMP1 (100pg/L)** | 0.46 (-1.38, 2.31) | 0.75 (-1.63, 3.12) | -0.13 (-1.94, 1.68) |
| **VCAM1 (1000000pg/L)** | 9.47 (-1.67, 20.6) | -13.5 (-24.8, -2.32) | -16.0 (-29.2, -2.78) |
| **CXCL10 (100pg/L)** | 1.20 (-1.13, 3.53) | -0.09 (-2.22, 2.04) | -1.05 (-3.33, 1.23) |
| **IL6 (10pg/L)** | -9.86 (-23.5, 3.82) | 2.43 (-2.92, 7.79) | 11.3 (-7.66, 30.2) |
| **MCSF (100pg/L)** | 12.1 (0.62, 23.5) | 0.72 (-15.3, 16.8) | -6.34 (-21.3, 8.59) |
| **Plasminogen Activator (100pg/L)** | 2.97 (-1.67, 7.60) | -4.26 (-10.5, 2.01) | -1.27 (-6.52, 3.99) |
| **VEGF (pg/L)** | 0.77 (-0.80, 2.33) | 0.23 (-1.31, 1.78) | -0.47 (-1.89, 0.95) |

* n=79 at baseline, n= 68 at changes at week4, and n= 63 at changes at week12

** COPD, Chronic Obstructive Pulmonary Disease

**Table S5. Radiological features of selected patients (N=48) with anti-tuberculosis and antiretroviral treatment**

|  | **Mean (Standard deviation)** |
| --- | --- |
| **Hard volume, mL** | 105 (142) |
| **Lung Total Glycolytic Activity** | 1270 (1530) |
| **Region Of Interest Volume in Computed Tomography, mL** | 67.8 (90.0) |
| **Maximum Standardized Uptake Value** | 9.8 (5.3) |
| **Percent lung affected, %** | 0.2 (0.2) |
