# Supplementary material for: Lung function and collagen 1a levels are associated with changes in 6 min walk test distance during treatment of TB among HIV-infected adults: a prospective cohort study
Source: BMC Pulm Med. 2023 Feb 3;23:53. doi: 10.1186/s12890-023-02325-7 (PMC9896708; doi:10.1186/s12890-023-02325-7)
Supplement: Supplementary file 2 — Additional file 2. Supplementary Figures. [file 12890_2023_2325_MOESM2_ESM.docx]

**Figure S1. Study Timeline**

**We enrolled ART naive adults with pulmonary TB and measured multiple plasma biomarkers of inflammation, along with lung function (e.g., FEV1, FVC) and COPD symptom assessment test at multiple time points after ART initiation out to a year. In addition, we performed PET-CTs to assess local inflammation at baseline and week 4 on ART. ART; anti-retroviral therapy, FEV1: forced expiratory volume in 1 second; FVC; forced vital capacity; COPD: chronic obstructive pulmonary disease**


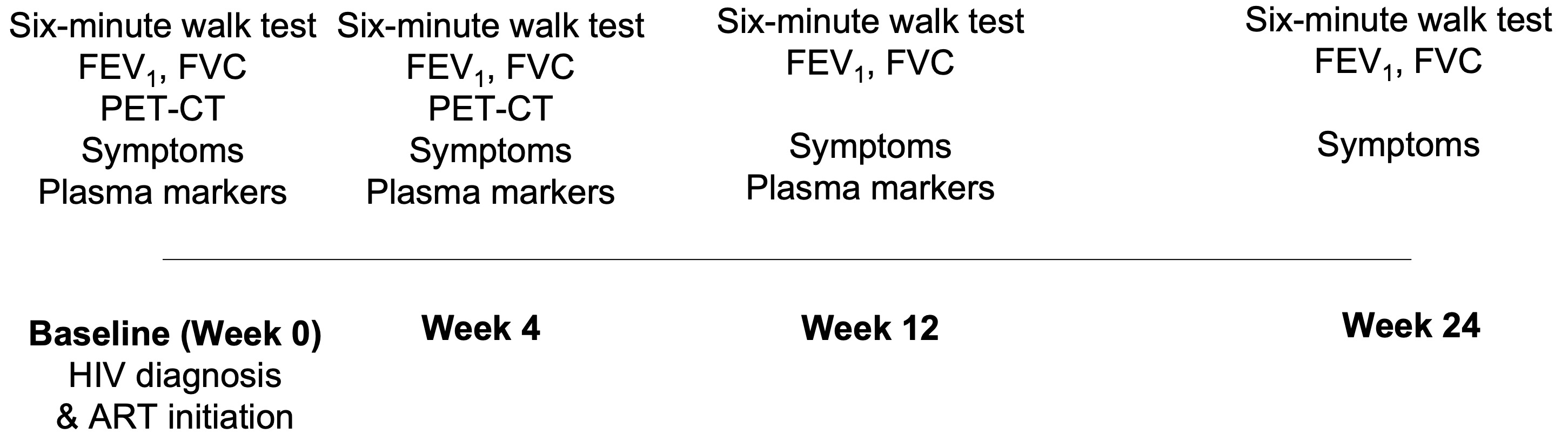


**Figure S2. Mean change in 6MWT distance, lung function and biomarker levels from baseline to week 24. The data for the 6MWT distance and lung function (FVC and FEV1) of participants up to week 24 and the plasma biomarker levels measured up to week 12 are presented. The grey lines represent the changes for n=89 individuals. The blue line represents the mean of changes and the red band represents the 95% confidence intervals.**


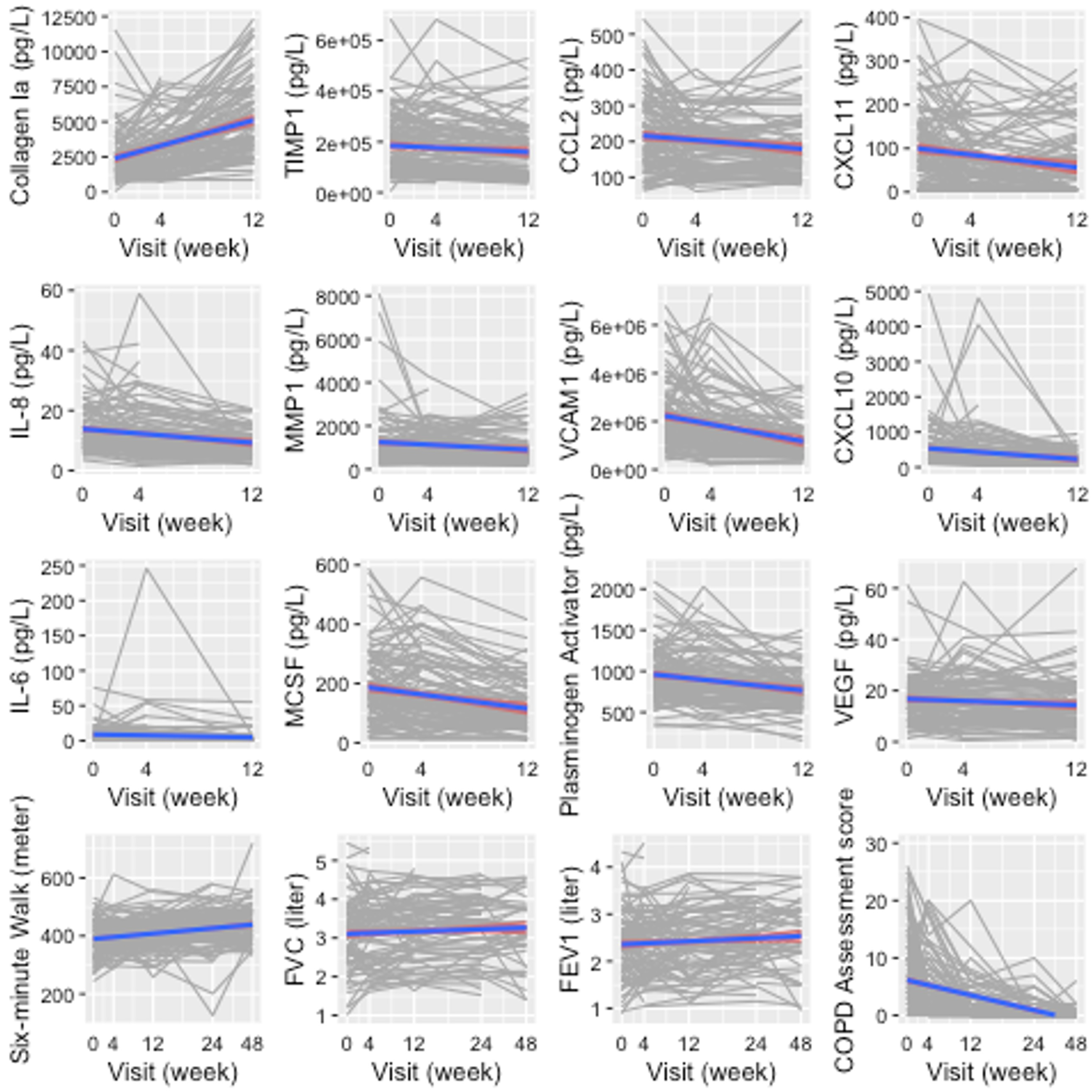


**Figure S3. A plot showing the correlation between clinical, radiologic factors and plasma biomarker levels. Numbers in each cell represent Pearson's correlation coefficient and only statistically significant correlation coefficients at p = 0.05 are presented. The red and blue colors represent the strength of the correlation with the thicker red = a stronger negative association and a thicker blue = a stronger positive association.**

**
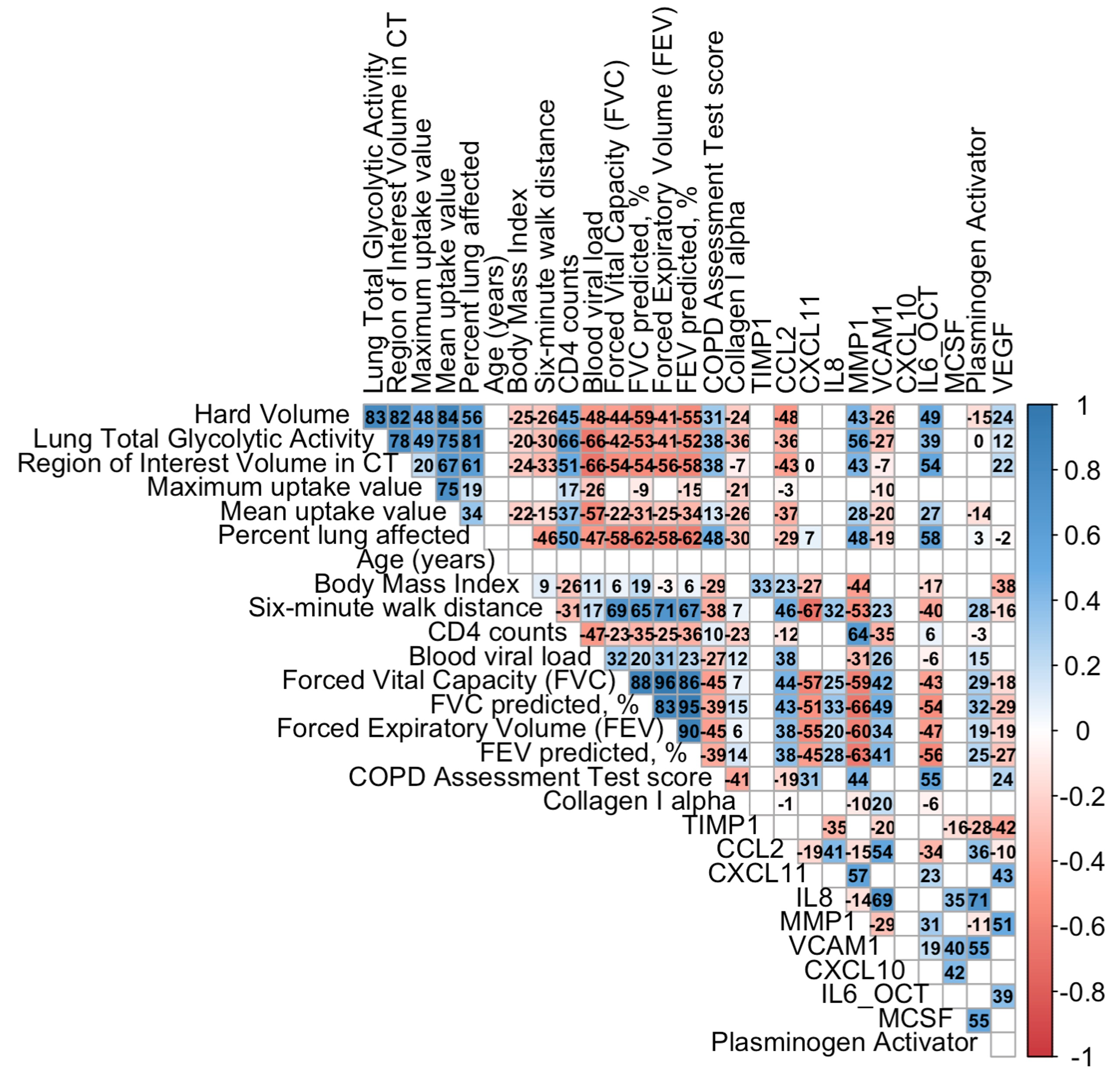
**
